# The Necessity for Endowing the Nursing Profession

**Published:** 1920-02-07

**Authors:** 


### THE MATRONS' AND SISTERS' DEPARTMENT.

### THE NECESSITY FOR ENDOWING THE NURSING PROFESSION.

Nursing has leaped of late years into an entirely new position. Formerly it was the luxury of the rich, a charity bestowed on the poor. It has now become a national necessity. It embraces all sorts and conditions of men. It is made up, as a profession, of all sorts and conditions of women. On the extent to which the members of the nursing services can be adequately prepared for their duties, on the spirit which informs them, on the discipline maintained among them, depends the future of the Empire. For it is not a feeble disease-ridden race which will be competent to deal with the problems which lie ahead.

Nurses as employees have no brilliant prospects. About five hundred a year may be regarded as the high-water mark of the profession. For the most part a competency is all that lies before the most successful nurse. They are not as a rule possessed of widespread influence. Their names are seldom known to the public outside their own circle of professional interest. It is clear that a body numbering many thousands of women of moderate incomes, unversed in public affairs, will be at a singular disadvantage when their collective interests are affected, unless they have set up a strong working organisation to speak and act for the whole body.

All the experience of the past tends to show that such a professional centre cannot be established and kept up to a high level of influence and power without a great deal of money. It must not depend solely on the subscriptions of members or it will either lack continuity or be tempted into the paths of popularity rather than of solid advancement. It must be able to secure the entire services of trained and educated women. It must have the means of alleviating distress among its members, of stimulating educational progress, of evoking widespread recognition by a series of schemes (all more or less costly to set in motion) for the benefit of various classes of nurses. It must be able to inspire spontaneous impulse and combined endeavour from nurses in every part of the kingdom. It must be in a position to command instant attention should it address remonstrances or representations to the more powerful departments with which its members' interests are linked. Its watchword must be that of the Musketeers of old, "One for all; all for one." In its rising importance, the importance of every member will increase. Leaning on its arm the friendless nurse will find herself protected, and the rapacious employer will cease from troubling.

This great work can only be carried through by a public well convinced of the need for organising the profession of nursing, guided by a council of professional nurses alive to the facts. Without public aid the Council of the College of Nursing can never achieve its aims. Without the College the purpose for which endowment is sought would never have been formulated. The fund which the *Daily Telegraph* is now generously lending its support to raise is thus a mark of cordial co-operation between the leaders of the profession and the public.

Do we fully realise the far-sighted policy which has brought about this coalition between public and professional interests? Let each nurse consider within herself her own reluctance to plead the cause of her sisters now in want, and that of the profession at large in the years to come, and acclaim the sound judgment which has given women no whit less jealous for the independence of nurses the courage to throw on the nation at large the burden, which is justly theirs, of endowing the nursing profession. It is because they recognise that nurses must be independent that they have dared to come down from the pedestal of independence on which some would fain see them immutably fixed.

There is very much more at stake in securing endowment than raising the benefit of individual nurses by support in adversity, by scholarship, or special opportunity. The purpose of this endowment is to raise the nursing profession from a disunited multitude of women, for the most part earning no more than suffices for a bare living, women individually unable to command consideration, to the status of a great united, well-ordered body, possessed of a voice which can be heard and a Centre from which they may exercise a powerful and health-giving influence in the affairs of the nation. Because the Council of the College has had this vision we predict their success, not merely in the immediate object of securing endowment, but in the greater purpose of turning it to account for the nation's good.

